# Effects of the herbal prescription Kami Guibi-tang on brain function in amnestic mild cognitive impairment: a task-based and resting-state fMRI study

**DOI:** 10.1007/s11682-026-01138-6

**Published:** 2026-03-23

**Authors:** Dongjoo Kim, Na-yeon Kim, Min-jae Kwak, Kyeong-Hwa Heo, Han-Gyul Lee, Seungwon Kwon, Seung-Yeon Cho, Seong-Uk Park, Woo-Sang Jung, Sang-Kwan Moon, Chang-Nam Ko, Hyug-Gi Kim, Geon-Ho Jahng, Jung-Mi Park

**Affiliations:** 1 Department of Cardiology and Neurology, Kyung Hee University College of Korean Medicine, Kyung Hee University Medical Center, Seoul, Republic of Korea; 2 Department of Clinical Korean Medicine, Graduate School, Kyung Hee University, Seoul, Republic of Korea; 3 Department of Cardiology and Neurology, College of Korean Medicine, Kyung Hee University, Seoul, Republic of Korea; 4 Stroke and Neurological Disorders Center, Kyung Hee University College of Korean Medicine, Kyung Hee University Hospital at Gangdong, Seoul, Republic of Korea; 5 Department of Radiology, Kyung Hee University Hospital, Kyung Hee University College of Medicine, Seoul, Republic of Korea; 6 Department of Radiology, Kyung Hee University Hospital at Gangdong, Kyung Hee University College of Medicine, Seoul, Republic of Korea

**Keywords:** Amnestic mild cognitive impairment, Kami Guibi-tang, fMRI, Functional connectivity, Memory task

## Abstract

Amnestic mild cognitive impairment represents a prodromal stage of Alzheimer’s disease characterized by episodic memory deficits and subtle alterations in brain function. Although the traditional herbal formula Kami Guibi-tang (KGT) has been reported to exert cognitive benefits, its neural effects have not been evaluated using functional neuroimaging. In this randomized, double-blind, placebo-controlled trial, 84 individuals with amnestic mild cognitive impairment received either KGT or placebo for 24 weeks. A total of 73 participants (36 in the KGT group and 37 in the placebo group) completed the intervention and were included in the final analyses. All participants underwent functional magnetic resonance imaging during a face-name association task and a working memory task, as well as resting-state scans. Task-based imaging data were analyzed using voxel-wise and region-of-interest approaches, and associations between changes in task-related activation and cognitive performance were also examined. Resting-state functional connectivity was analyzed to examine regional connectivity changes. At baseline, the placebo group showed greater activation in memory- and attention-related regions, including the posterior cingulate cortex, frontal areas, and thalamus. These voxel-wise differences were no longer observed after the intervention. Region-of-interest analyses using linear mixed-effects models revealed significant group × time interactions in several frontal, cingulate, and thalamic regions, with activation declining in the placebo group but remaining relatively preserved or showing modest increases in the KGT group. Resting-state analyses further demonstrated increased connectivity between the left precuneus and right posterior cingulate cortex following KGT administration. These findings suggest that KGT may modulate brain activity and connectivity in regions involved in episodic and working memory. While preliminary, these results provide neuroimaging-based evidence for its potential effects on memory-related neural processes in the early stages of cognitive decline.

**Supplementary Information:**

The online version contains supplementary material available at 10.1007/s11682-026-01138-6.

## Introduction

Mild cognitive impairment (MCI) is characterized by impairments in cognitive functions while preserving the ability to perform independent daily activities (Wang et al., 2021). MCI can be subcategorized into amnestic MCI (aMCI) and non-amnestic MCI (non-aMCI). Memory impairment is the predominant symptom of aMCI and is often accompanied by deficits in language and executive function (Kasper et al., 2020). aMCI is regarded as a prodromal stage of Alzheimer’s disease (AD), with a significant proportion of aMCI cases progressing to AD. Approximately 10–15% of individuals with aMCI develop AD annually, and up to 80% transition to AD within six years, compared to only 1–2% of cognitively normal individuals developing AD each year (Petersen et al., 1999; Shin et al., 2015). As no treatments currently exist to fundamentally alter the progression of AD, aMCI represents a critical window for therapeutic interventions.

Recently, the FDA approved two passive amyloid-β (Aβ) immunotherapies, lecanemab (Leqembi^®^) and donanemab, for early AD, including aMCI with confirmed Aβ pathology (Cummings et al., 2023). These treatments primarily slow cognitive decline rather than reverse it and are more effective in clearing amyloid aggregates than improving cognition (Van Dyck et al., 2023). Furthermore, adverse effects such as amyloid-related imaging abnormalities (ARIA), which can include cerebral edema or microhemorrhages, their high cost, and limited clinical efficacy highlight the need for the development of alternative therapeutic approaches (Salloway et al., 2022; Söderberg et al., 2023). In this context, Korean medical interventions, including acupuncture and herbal medicine, have shown potential benefits, such as reducing the conversion rate to dementia and improving cognitive test performance (Kim et al., 2019; Shin et al., 2021; Tian et al., 2017; Xingjie et al., 2023; Zhang et al., 2019).

Kami Guibi-tang (KGT; Kami-guibi-tang in Korean, Jia-wei-gui-pi-tang in Chinese, and Kami-kihi-to in Japanese) is a traditional herbal formula that has been used for centuries in East Asia to treat amnesia (Watari et al., 2015), insomnia (Lee et al., 2018), and loss of appetite (Wu & Leonard, 2019). Numerous studies conducted in vivo, in vitro, and in clinical settings have demonstrated the cognitive function-enhancing effects and several potential mechanisms have been suggested. A clinical study reported that oral administration of KGT granules improved the Mini-Mental State Examination (MMSE) scores of patients with AD, with notable improvements in orientation and attention (Higashi et al., 2007). Additionally, combination therapy consisting of KGT and donepezil for 9 months improved the MMSE score of AD patients compared to the administration of donepezil alone (Ishida, 2016). Although several mechanisms—such as calcium influx inhibition and tau dephosphorylation—have been proposed, primarily based on in vitro neuronal studies, the precise pathways through which KGT exerts its cognitive effects are not yet fully understood (Tohda et al., 2008; Watari et al., 2014). Despite promising preclinical and clinical findings, no studies have investigated the effects of KGT using functional magnetic resonance imaging (fMRI) to assess brain activation and connectivity.

Episodic memory impairment is one of the hallmark features of aMCI (Tulving, 2002), and fMRI studies have consistently implicated functional abnormalities in brain regions such as the medial temporal lobe (Dickerson et al., 2004), hippocampus (Bai et al., 2009), and default mode network (DMN) (Dunn et al., 2014). Working memory is also frequently impaired in aMCI and has been shown to predict progression to AD (Brandt et al., 2009; Klekociuk et al., 2014; Saunders & Summers, 2011). These cognitive domains are supported by prefrontal and medial temporal circuits, which are vulnerable to early AD pathology.

In addition to task-related dysfunctions, resting-state functional connectivity (FC) studies have revealed disrupted synchronization within large-scale brain networks in aMCI. Notably, reduced connectivity between core regions of the DMN—such as the posterior cingulate cortex (PCC) and precuneus—has been repeatedly observed in patients with aMCI and is considered a sensitive biomarker for early network degeneration (Dunn et al., 2014; Greicius et al., 2004; Wang et al., 2006). As the DMN is tightly linked to self-referential processing and episodic memory retrieval, interventions that restore or enhance DMN connectivity may provide therapeutic benefits in the prodromal stages of AD.

Therefore, assessing the effects of a drug on both episodic and working memory, along with underlying brain function, is crucial for evaluating its therapeutic potential for aMCI and elucidating its mechanism of action.

Based on prior evidence, we aimed to explore whether KGT modulates brain activation and functional connectivity within memory- and attention-related regions in patients with aMCI, thereby identifying potential neural mechanisms preceding or accompanying cognitive changes. To test this, we conducted a randomized, placebo-controlled trial using task-based and resting-state fMRI.

## Methods

### Ethics and design

This study was part of a larger randomized controlled trial designed to evaluate the effects of KGT on cognitive function and brain activity. The present study focused exclusively on the neuroimaging component. The trial adhered to the principles of the Declaration of Helsinki and the Korean Good Clinical Practice (KGCP) guidelines. Ethical approval was obtained from the Institutional Review Board of Kyung Hee University Hospital at Gangdong (KHNMC-OH-IRB No. 2021-12-004-002) and the Korean Ministry of Food and Drug Safety (approval number: 32756). The study protocol was registered at the Clinical Research Information Service (CRIS) on February 24, 2022 (registration number: KCT0007039, http://cris.nih.go.kr/). Written informed consent was obtained from all participants before enrollment.

### Participants

Participants were recruited between March 2022 and March 2024 from Kyung Hee University Hospital at Gangdong. Eligible individuals who had subjective memory complaints and met the following criteria were included: (1) diagnosis of aMCI by a neurologist, with objective cognitive impairment measured using the SNSB-D, a Global Deterioration Scale (GDS) score of 3, a Clinical Dementia Rating (CDR) of 0.5, and inclusion of both single-domain and multiple-domain aMCI cases; (2) aged between 55 and 90 years; (3) no use of cognitive-enhancing drugs within the past two weeks; (4) stable medication regimen for chronic conditions; (5) intact communication ability; and (6) no contraindications or disqualifying conditions related to imaging procedures.

Key exclusion criteria included: (1) dementia diagnosis; (2) neurological disorders other than cognitive dysfunction; (3) major neurodegenerative diseases; (4) cerebral abnormalities resulting from hypoxemia, vitamin deficiencies, infectious diseases, brain tumors, endocrine-metabolic disorders, or intellectual disability; (5) clinical evidence of cerebrovascular disease, or suspected territorial infarcts or multiple strokes on MRI; (6) seizure history (except febrile seizures); (7) history or presence of major depression; (8) psychiatric illness requiring antipsychotics; (9) substance abuse; (10) life-threatening conditions requiring immediate treatment; and (11) considered unsuitable for participation at the discretion of the investigator. The full list of exclusion criteria is provided in Supplementary Information S1.

### Randomization and blinding

Qualifying participants were randomly assigned to the KGT or placebo group in a 1:1 ratio using block randomization by an independent investigator. KGT and placebo granules were manufactured to be identical in appearance and physical properties. In accordance with the randomization table, the manufacturer packaged the study drugs identically, labeled them with serial numbers. An independent pharmacist managed drug allocation based on enrollment order. All participants, pharmacists, investigators, and outcome assessors remained blinded throughout the trial.

### Interventions

KGT or placebo granules (3.0 g per dose) were administered orally with water three times daily, 30 min post-meal, for 24 weeks. Both KGT and placebo were manufactured by Hanpoong Pharmaceutical Co., Ltd. (Seoul, South Korea), in compliance with Good Manufacturing Practice (GMP) standards. KGT consisted of a yellow-brown mixture of spray-dried hot water extracts from 15 medicinal herbs, with detailed composition and dosage provided in Table 1. The placebo consisted of corn starch, lactose, hydroxypropyl cellulose, caramel color, tartrazine (FD&C Yellow 5), Allura Red AC (FD&C Red 40), and Ssanghwa flavor, formulated to match the KGT in appearance and taste. A 24-week supply was dispensed in two installments (12 weeks each). At each follow-up, unused doses were returned and documented, and compliance was calculated as the proportion of prescribed doses actually taken. Participants with compliance below 80% were excluded from the final analysis. Medications for stable chronic conditions were allowed; however, drugs affecting cognition were prohibited.

**Table 1:** Composition of Kami Guibi-tang granules

Scientific name	Herbal name	Part used	Dosage (g)
*Panax ginseng* C. A. Meyer	Ginseng Radix	Root	1
*Atractylodes macrocephala* Koidz	Atractylodis Rhizoma Alba	Rhizome	1
*Poria cocos* Wolf	Poria	Sclerotium	1
*Astragalus membranaceus* Bunge	Astragali Radix	Root	1
*Dimocarpus longan* Lour	Longan Arillus	Arill	1
*Zizyphus jujuba Miller* var. *spinosa* Hu ex H. F. Chou	Zizyphi Spinosi Semen	Seed	1
*Bupleurum falcatum* Linné	Bupleuri Radix	Root	1
*Angelica gigas* Nakai	Angelicae Radix	Root	0.67
*Polygala tenuifolia* Willdenow	Polygalae Radix	Root	0.67
*Gardenia jasminoides* J.Ellis	Gardeniae Fructus	Fruit	0.67
*Paeonia suffruticosa* Andrews	Moutan Radicis Cortex	Rhizodermis	0.67
*Zizyphus jujuba Miller var. inermis* Rehder	Zizyphi Fructus	Fruit	0.67
*Aucklandia lappa* Decne	Aucklandiae Radix	Root	0.33
*Glycyrrhiza uralensis* Fischer	Glycyrrhizae Radix	Root	0.33
*Zingiber officinale* Roscoe	Zingiberis Rhizoma	Rhizome	0.33

### Assessments

All participants were assessed for outcome measures at baseline and after 24 weeks of intervention. In the parent randomized controlled trial, the primary clinical endpoint was the change in the SNSB-D total score at 24 weeks. However, the present manuscript reports on the neuroimaging component of that trial, which was designed as an exploratory study focusing on fMRI measures. Accordingly, the endpoints of this study were the changes in task-related brain activation and resting-state functional connectivity, while SNSB-D domain scores were used only for exploratory correlation analyses with imaging data.

#### Image acquisition

MRI scans were acquired using a 3T scanner (Ingenia, Philips Medical Systems, Netherlands). The imaging protocol included sagittal 3D T1-weighted turbo field echo (TFE), as well as BOLD-based fMRI encompassing resting-state and task-based runs.

High-resolution anatomical 3D T1-weighted images were acquired for functional data co-registration (TR/TE = 9.9/4.6 ms; flip angle = 8°; FOV = 240 × 240 × 190 mm³; matrix = 240 × 240; voxel size = 1 × 1 × 1 mm³; 190 slices; scan time = 5 min).

Functional images were collected using T2*-weighted EPI (TR/TE = 2000/30 ms; flip angle = 70°; FOV = 220 × 220 × 150 mm³; acquisition matrix = 72 × 70; reconstruction matrix = 80; acquisition voxel size = 3.06 × 3.14 × 3.00 mm³; reconstruction voxel size = 2.75 × 2.75 × 3.00 mm³; slice thickness = 3 mm; 50 slices; SENSE factor = 2; EPI factor = 35; 180 volumes; total scan duration = 18 min across three 6-minute runs).

During resting-state acquisition, participants were instructed to keep their eyes closed while remaining awake. All scans were reviewed by an independent radiologist, confirming no structural brain abnormalities.

#### fMRI tasks

**Fig. 1:**
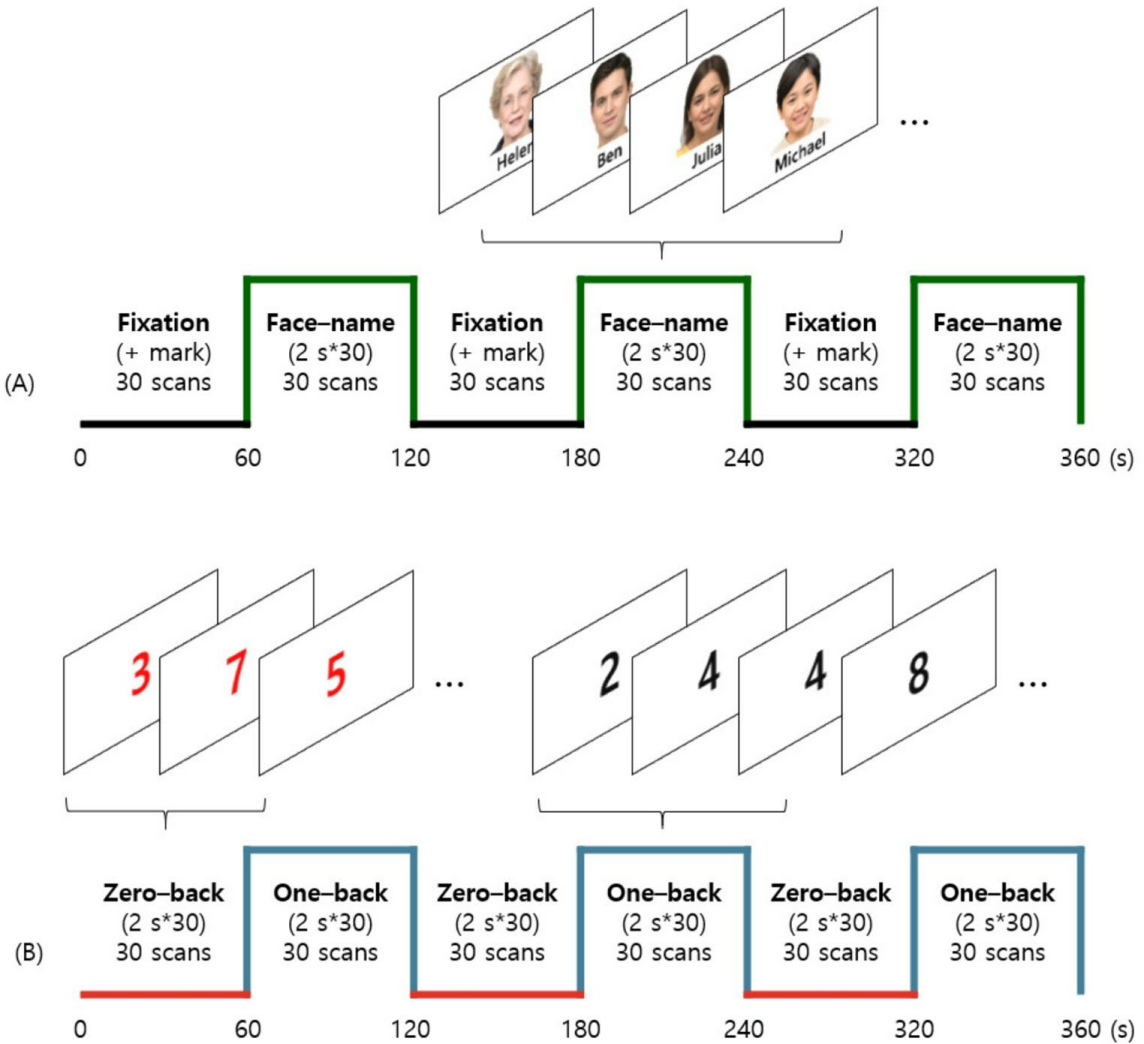
Task design for fMRI. **A** The face-name association memory task. **B** The working memory N-back task. Repetition time = 2s, Number of volumes = 180, Number of slices = 32, Runtime = 6 min

Two block-designed fMRI tasks were performed after the resting scan: the face-name association task, which evaluates verbal and non-verbal memory encoding processes (Klamer et al., 2017), and the N-back task, which assesses working memory (Stopford et al., 2012). Each task followed a block design and lasted 6 min, during which 180 volumes were acquired (TR = 2000 ms) (Fig. 1). All participants received a pre-scan briefing and completed practice sessions.

The face-name association task alternated between three 1-minute fixation blocks and three 1-minute encoding blocks. During fixation blocks, a black cross was displayed on a gray background. During encoding blocks, participants were shown 30 unique face-name pairs (2 s each) and instructed to memorize the associations. Importantly, the task was designed as an encoding-only paradigm; no in-scanner or post-scan recall phase was administered. Thus, behavioral measures reflecting successful versus unsuccessful encoding were not available, and the paradigm was used solely to assess encoding attempt–related activation.

The N-back task consisted of alternating 1-minute blocks of 0-back and 1-back conditions, repeated three times. In the 0-back blocks, participants pressed a handheld button if a designated digit (“7”) appeared among randomly presented red digits, serving as an attention baseline condition. In the 1-back blocks, they responded when the current black digit matched the digit immediately preceding it, requiring maintenance and comparison of recent stimuli (working memory load). Task performance, including response accuracy and reaction time during the N-back task, was analyzed.

All tasks were visually presented using MR-compatible equipment and displayed via a screen and mirror mounted on the head coil. Participant responses were recorded using a response pad.

#### Neuropsychological measures

Neuropsychological assessments were conducted at baseline and after the 24-week intervention. Global cognitive function was evaluated using the Korean version of the MMSE (K-MMSE) to characterize baseline and post-intervention cognitive status. The SNSB-D was also administered to assess domain-specific cognitive function; however, in this neuroimaging-focused study, SNSB-D domain scores were used solely for exploratory correlations with fMRI activation and were not reported as formal clinical endpoints. The composition and scoring system of the SNSB-D domains are detailed in Supplementary Table S1.

### fMRI analysis

#### Task-based fMRI analysis

##### Preprocessing

Task-based fMRI data were preprocessed and analyzed using SPM12 (Wellcome Department of Cognitive Neurology, Institute of Neurology, London, UK). The main preprocessing steps for task-based functional data included: (1) slice timing correction to minimize discrepancies in acquisition timing across scanning layers; (2) realignment to correct for head motion; (3) coregistration to align functional images into 3D structural images; (4) segmentation to divide the 3D structural image into gray matter, white matter, and cerebrospinal fluid; (5) normalization to the Montreal Neurological Institute (MNI) space with resampling to a voxel size of 2 × 2 × 2 mm³; and (6) spatial smoothing using a Gaussian kernel of 7 × 7 × 7 mm³ full width at half maximum (FWHM) to reduce residual anatomical variability and increase the signal-to-noise ratio.

All participants met the predefined head motion thresholds (< 3 mm translation and < 3° rotation), and no volumes or participants were excluded due to excessive motion artifacts.

##### Voxel-based whole-brain analysis

At the individual level, a generalized linear model (GLM) was applied to estimate the task-related BOLD responses. Contrast maps were generated for each task condition: (1) fixation > face-name and fixation < face-name, and (2) 0-back > 1-back and 0-back < 1-back. These maps were entered into second-level random effects analyses to examine group-level differences. Paired t-tests were used for within-group comparisons and two-sample t-tests were used for between-group comparisons, with age as a covariate.

The voxel-based results did not remain significant after FDR correction; therefore, for exploratory purposes, statistical significance was defined as uncorrected *p* < 0.001 with a cluster extent threshold of 50 voxels. All coordinates were reported in the MNI space, and brain regions were localized using Brodmann areas.

##### Region of Interest (ROI) analysis

ROIs were defined using two approaches. First, functionally defined cluster-based ROIs were derived from significant baseline activation clusters identified in the voxel-wise analysis. Specifically, during the face–name association task, a right PCC cluster showed greater baseline activation in the placebo group than in the KGT group; during the N-back task, nine discrete clusters were identified across frontal, cingulate, and thalamic regions, which likewise exhibited greater baseline activation in the placebo group than in the KGT group. These task-specific clusters were used as cluster-based ROIs in subsequent analyses. Second, atlas-based ROIs were selected based on prior literature implicating these regions in episodic and working memory processes, including the hippocampus (Yonelinas et al., 2024), parahippocampal gyrus (PHG) (Li et al., 2016), dorsolateral prefrontal cortex (DLPFC) (Barbey et al., 2013), ventrolateral prefrontal cortex (VLPFC) (Segal & Elkana, 2023), precuneus (Flanagin et al., 2023), and PCC (Huo et al., 2018; Wang et al., 2019). The left and right hemispheres were analyzed separately. For clarity, ROIs with overlapping anatomical labels (e.g., PCC) are denoted as “<region> (cluster-based ROI)” when derived from voxel-based analyses.

ROI signal intensity was defined as the mean parameter estimates (β values) extracted from the individual first-level contrast maps (face-name > fixation, 0-back > 1-back) within each predefined ROI mask. These values represent the average task-related activation across all voxels within each ROI.

Group × time effects on ROI signal intensity were examined using linear mixed-effects models (LMMs), with age and hypertension included as covariates. To examine associations between changes in ROI activation and changes in SNSB domain scores, linear regression analyses were performed, adjusting for age and hypertension (*p* < 0.05).

#### Resting state fMRI analysis

##### Preprocessing

For the resting-state data, the first 10 volumes were discarded to allow for magnetic field stabilization. The remaining preprocessing pipeline included: (1) slice timing correction; (2) realignment; (3) spatial normalization; (4) spatial smoothing with a Gaussian kernel of 6 × 6 × 6 mm³ FWHM; (5) regression of nuisance covariates to remove non-neuronal signals, including white matter, cerebrospinal fluid, and six motion parameters; and (6) temporal band-pass filtering (0.01–0.08 Hz) to isolate low-frequency blood-oxygen-level-dependent (BOLD) fluctuations. The preprocessing pipeline and related parameter settings were based on the recommendations of the REST toolkit (Song et al., 2011), which has been widely adopted in resting-state fMRI research.

##### Seed-based functional connectivity

Resting-state seed-based FC analysis was conducted using RESTplus (version 1.31, www.restfmri.net) (Jia et al., 2019). For resting-state analyses, cluster-based ROIs were defined based on the voxel-based task analyses: regions showing significantly greater baseline activation during the face-name task were defined as Cluster #1, and those during the N-back task were defined as Cluster #2. For each ROI, the mean BOLD signal time series was obtained and subjected to linear detrending and band-pass filtering (0.01–0.08 Hz) to isolate low-frequency fluctuations. A correlation matrix (Pearson’s r) was computed for all of the ROI pairs. Fisher’s r-to-z transformation was applied to normalize the correlation values.

### Statistical analysis

The sample size was calculated based on a previous pilot study that assessed changes in the total SNSB-D Score between KGT and placebo groups (Shin et al., 2021). The effect size of 0.698 was derived by dividing the mean difference in the total SNSB-D Score between the two groups after 24 weeks (14.11) by the pooled standard deviation (20.2). Considering this effect size, a power of 80% (α = 0.05, β = 0.20), a 20% dropout rate, and a 1:1 allocation ratio, the required sample size was estimated to be 42 participants per group (total *n* = 84). In subsequent analyses, hypertension status was included as a covariate because it was the only baseline variable showing a significant difference between the groups.

All statistical analyses were performed using R (version 4.1.2). Categorical variables, such as sex and hypertension status, were analyzed using the chi-squared or Fisher’s exact tests. Continuous variables, including age and education, were analyzed using independent t- or Wilcoxon rank-sum tests depending on normality assumptions.

For repeated-measures outcomes—including ROI signal intensity, N-back task performance (accuracy and reaction time), and K-MMSE scores—group × time effects were examined using LMMs. Group, time, and their interaction were entered as fixed effects, and participant-level random intercepts were modeled to account for within-subject correlation. Age and hypertension status were included as covariates. LMM results were reported as β coefficients with 95% confidence intervals and p-values. All statistical tests were two-sided, with a significance threshold of *p* < 0.05.

## Results

### Demographic and participant characteristics

**Fig. 2:**
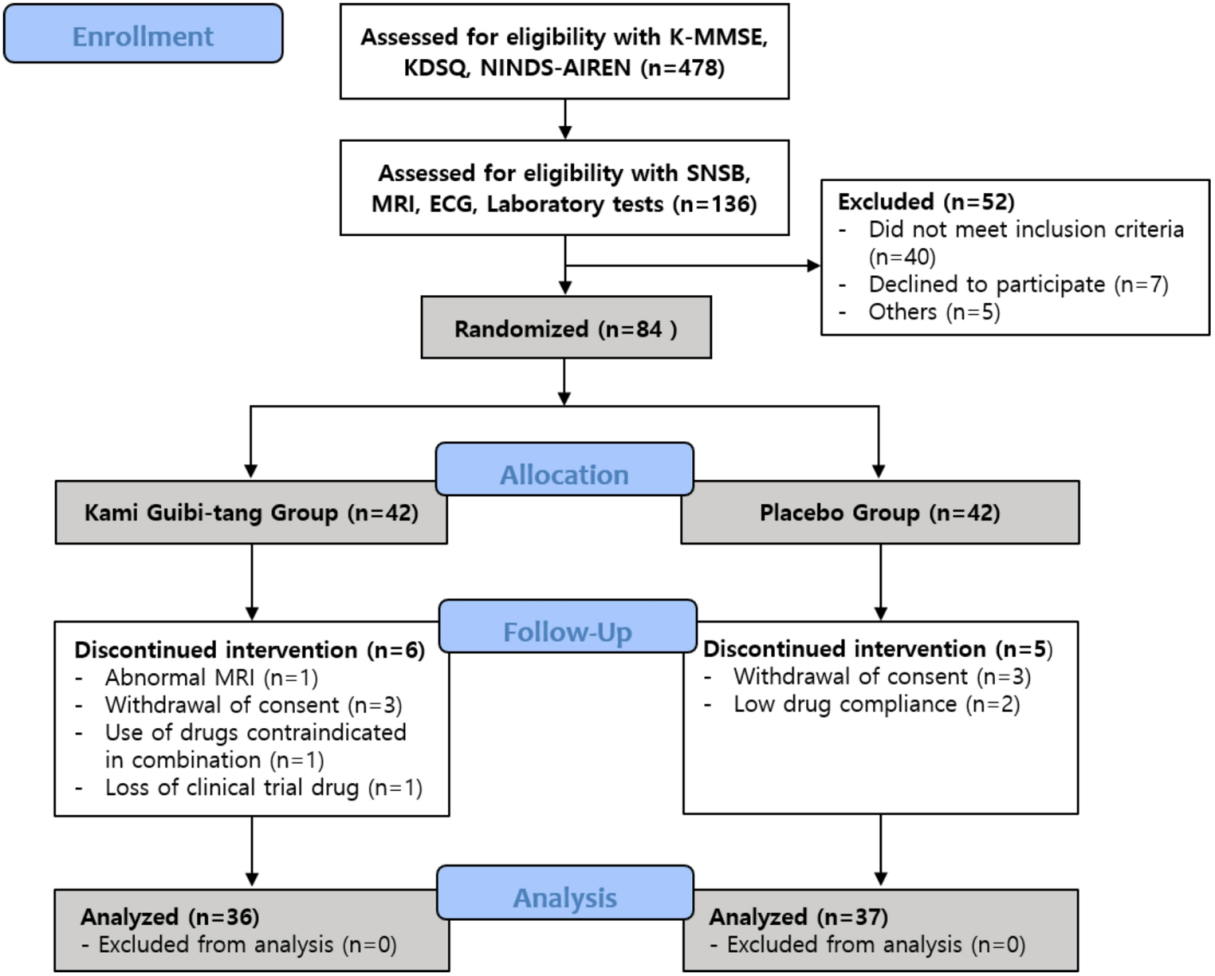
Study flow diagram. K-MMSE, Korean version Mini-Mental State Examination-2; KDSQ, Korean Dementia Screening Questionnaire; NINDS-AIREN, National Institute of Neurological Disorders and Stroke-Association Internationale pour la Recherche et l’Enseignement en Neurosciences; SNSB, Seoul Neuropsychological Screening Battery Dementia version; MRI, Magnetic resonance imaging; ECG, Electrocardiogram

A total of 478 individuals were initially screened, of which 84 eligible participants were randomized into the KGT group (*n* = 42) or placebo group (*n* = 42). During the follow-up period, 6 participants in the KGT group and 5 in the placebo group discontinued the intervention because of abnormal MRI findings, withdrawal of consent, or low drug compliance. Ultimately, 36 participants from the KGT group and 37 from the placebo group completed the study and were therefore included in the final analysis (Fig. 2).

The demographic and baseline clinical characteristics of the participants are presented in Table 2. No significant group differences were observed in age, sex, or years of education. However, hypertension was significantly more prevalent in the KGT group than in the placebo group (*p* = 0.004). Although not statistically significant, the KGT group tended to be older, and both groups showed a predominance of male participants.

**Table 2:** Demographic and baseline characteristics of the study participants

Characteristics	KGT group (*n* = 36)	Placebo group (*n* = 37)	*p*-value^*^
Age	74.89 ± 6.05	72.43 ± 7.3	0.123
Male	23 (63.9)	27 (73.0)	0.406
Education years	14.08 ± 3.62	13.62 ± 4.44	0.595
Height (cm)	161.52 ± 9.12	163.51 ± 8.65	0.342
Weight (kg)	61.61 ± 8.7	64.61 ± 13.35	0.261
Hypertension	17 (47.2)	6 (16.2)	**0.004**

Continuous variables are presented as the mean ± standard deviation and categorical variables as numbers (percentages). Values in bold indicate statistical significance (*p*-value < 0.05)

^*^p-values were obtained using the two-sample t-test, Wilcoxon rank sum test, or chi-square test, as appropriate

### Voxel-based whole-brain analysis

In both the KGT and placebo groups, no brain regions showed significant within-group changes in task-related functional activation above the statistical threshold for either the face-name or N-back tasks.

**Fig. 3:**
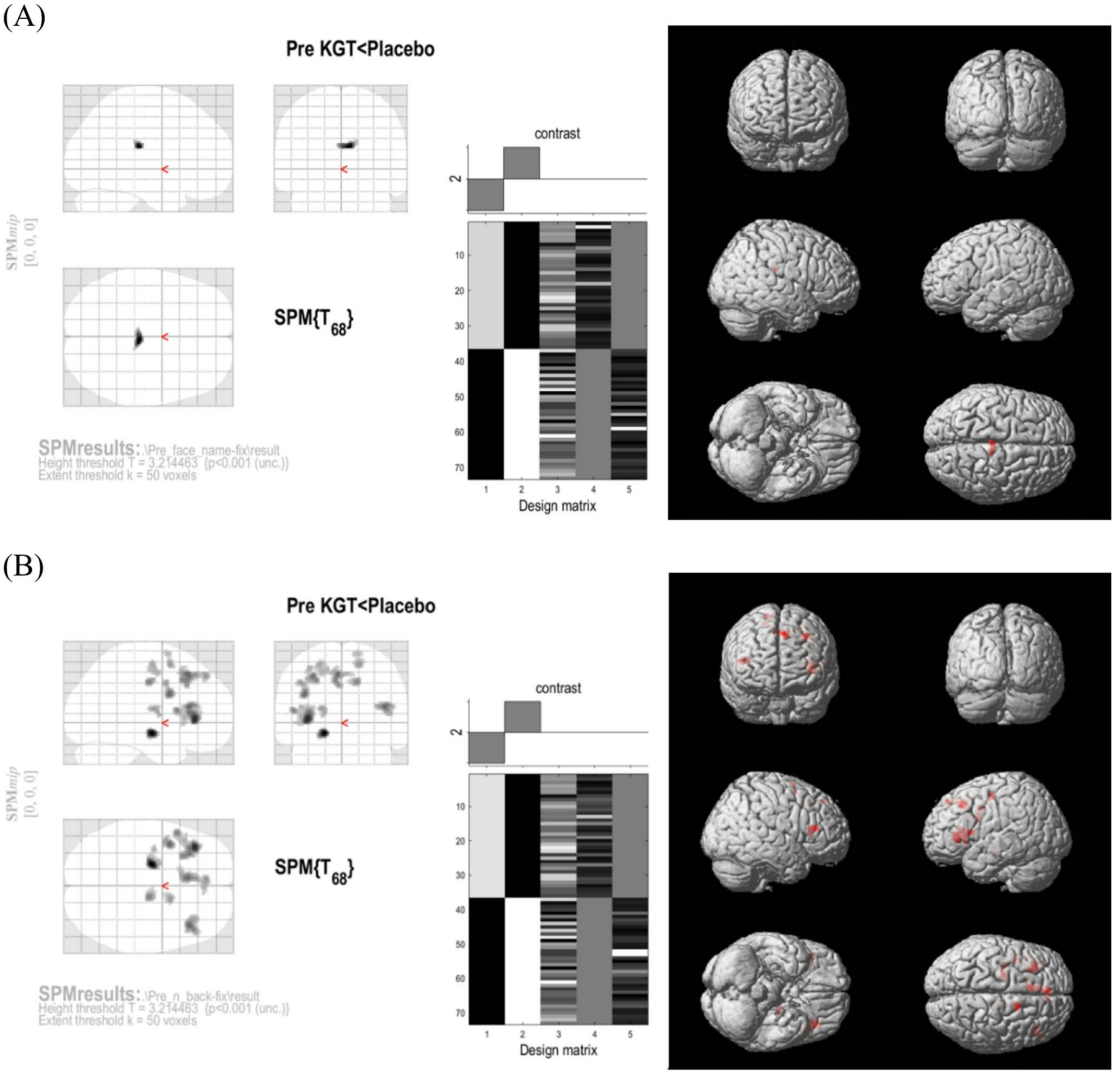
Voxel-based whole-brain analysis at baseline: Regions showing greater activation in the placebo group compared to the KGT group during (**A**) the face-name task and (**B**) the N-back task. (uncorrected *p* < 0.001, cluster size ≥ 50 voxels). KGT, Kami Guibi-tang; SPM, Statistical Parametric Mapping

In the face-name task, between-group analysis revealed significantly greater activation in the placebo group than in the KGT group in the right PCC at baseline (*p* < 0.001; Fig. 3A, Table 3). However, no significant differences were observed between the groups at 24 weeks. For the N-back task, the placebo group also showed significantly greater activation than the KGT group at baseline, with multiple clusters identified in the frontal, cingulate, and subcortical regions (*p* < 0.001; Fig. 3B, Table 3). As with the face-name task, these differences were no longer present at 24 weeks. These task-specific clusters (Fig. 3, Table 3) were subsequently defined as cluster-based ROIs and included in the ROI analysis, in addition to the predefined atlas-based ROIs.

**Table 3:** Significant between-group activation clusters identified by voxel-based analysis at baseline (*p* < 0.001 uncorrected, cluster size ≥ 50 voxels)

Region		Cluster size	MNI coordinates			BA	T-value
			X	Y	Z		
Face-name task: KGT < Placebo at baseline							
Rt	Posterior Cingulate	57	4.23	–27.76	19.74	23	3.9
Rt	Posterior Cingulate		11.56	–32.05	24.86	23	3.27
N-back task: KGT < Placebo at baseline							
Lt	Lentiform Nucleus	80	–19.42	–11.8	–7.98		5.02
Lt	Inferior Frontal Gyrus	382	–34.28	28.04	8.16	45	4.62
Lt	Precentral Gyrus		–47.38	8.78	13.32	44	3.9
Lt	Precentral Gyrus		–41.74	16.73	8.76	44	3.6
Lt	Medial Frontal Gyrus	272	–7.06	16.61	47.17	6	4.14
Lt	Superior Frontal Gyrus		–6.99	33.56	46.98	8	3.77
Lt	Superior Frontal Gyrus		0.36	31.31	50.49	8	3.6
Lt	Precentral Gyrus	108	–38.33	1.75	27.22	6	4.12
Lt	Precentral Gyrus		–42.18	–3	37.51	6	3.74
Lt	Middle Frontal Gyrus	119	–29.26	18.59	46.98	8	4.06
Rt	Cingulate Gyrus	66	11.45	–20.05	36.8	24	3.9
Rt	Medial Frontal Gyrus	82	13.12	–1.31	56.62	6	3.77
Rt	Superior Frontal Gyrus		11.14	–4.03	65.34	6	3.51
Lt	Thalamus	57	–21.55	–12.02	13.58		3.72
Lt	Precentral Gyrus	161	–23.94	–17.88	54.43	6	3.64
Lt	Precentral Gyrus		–33.12	–15.45	49.1	4	3.59
Lt	Cingulate Gyrus		–21.97	–13.29	45.89	24	3.57

*MNI* Montreal Neurological Institute, *BA* Brodmann area, *Lt* left, *Rt* right

### ROI-based analysis

A summary of ROIs that reached statistical significance in the LMM group × time interaction is shown in Table 4, with detailed results provided in Supplementary Tables S2 and S3. Linear mixed-effects models identified significant interaction effects in several regions. During the face–name association task, a significant interaction emerged in the right PCC (cluster-based ROI) (β = 0.524, 95% CI = 0.056–0.992, *p* = 0.029), reflecting a relative preservation of activation in the KGT group, in contrast to a marked decline in the placebo group over 24 weeks.

**Table 4:** ROI activation showing significant group × time interactions

ROI	KGT group (*n* = 36) (mean ± SD)		Placebo group (*n* = 37) (mean ± SD)		ΔKGT mean	ΔPlacebo mean	β (Group×Time)	95% CI for β	*p* for Group x Time
	Baseline	24th week	Baseline	24th week					
Face-name association task									
PCC_Rt. (cluster-based)	–0.168 ± 0.57	–0.112 ± 0.70	0.314 ± 1.10	–0.162 ± 0.54	0.048	–0.476	0.524	0.056, 0.992	**0.029**
N-back task									
MFG_Lt.	–0.082 ± 0.33	0.018 ± 0.21	0.171 ± 0.41	0.033 ± 0.38	0.1	–0.138	0.238	0.013, 0.463	**0.039**
Cingulate Gyrus_Rt.	–0.066 ± 0.31	0.017 ± 0.2	0.189 ± 0.38	0.005 ± 0.33	0.084	–0.184	0.267	0.064, 0.471	**0.011**
MeFG_Rt.	0.019 ± 0.32	0.081 ± 0.3	0.306 ± 0.51	0.109 ± 0.38	0.062	–0.197	0.259	0.039, 0.480	**0.022**
Thalamus_Lt.	0.018 ± 0.26	0.035 ± 0.19	0.211 ± 0.27	0.048 ± 0.28	0.018	–0.163	0.181	0.014, 0.348	**0.035**
Precentral Gyrus/Cingulate_Lt.	–0.02 ± 0.32	0.043 ± 0.21	0.209 ± 0.33	0.043 ± 0.3	0.063	–0.166	0.229	0.041, 0.417	**0.018**

Statistical significance was evaluated using linear mixed-effects models assessing group × time interaction effects. Only ROIs showing significant interaction effects (*p* < 0.05; bolded values) are presented. Full model results are provided in Supplementary Tables S2 and S3

*ROI* Region of interest, *KGT* Kami-guibi-tang, *PCC* posterior cingulate cortex, *MFG* middle frontal gyrus, *MeFG* medial frontal gyrus, *Lt* left, *Rt* right

During the N-back task, significant group × time interactions were likewise observed in multiple frontal, cingulate, and subcortical regions. These included the left MFG (β = 0.238, 95% CI = 0.013–0.463, *p* = 0.039), right cingulate gyrus (β = 0.267, 95% CI = 0.064–0.471, *p* = 0.011), right medial frontal gyrus (β = 0.259, 95% CI = 0.039–0.480, *p* = 0.022), left thalamus (β = 0.181, 95% CI = 0.014–0.348, *p* = 0.035), and the left precentral/cingulate cluster (β = 0.229, 95% CI = 0.041–0.417, *p* = 0.018).

**Fig. 4:**
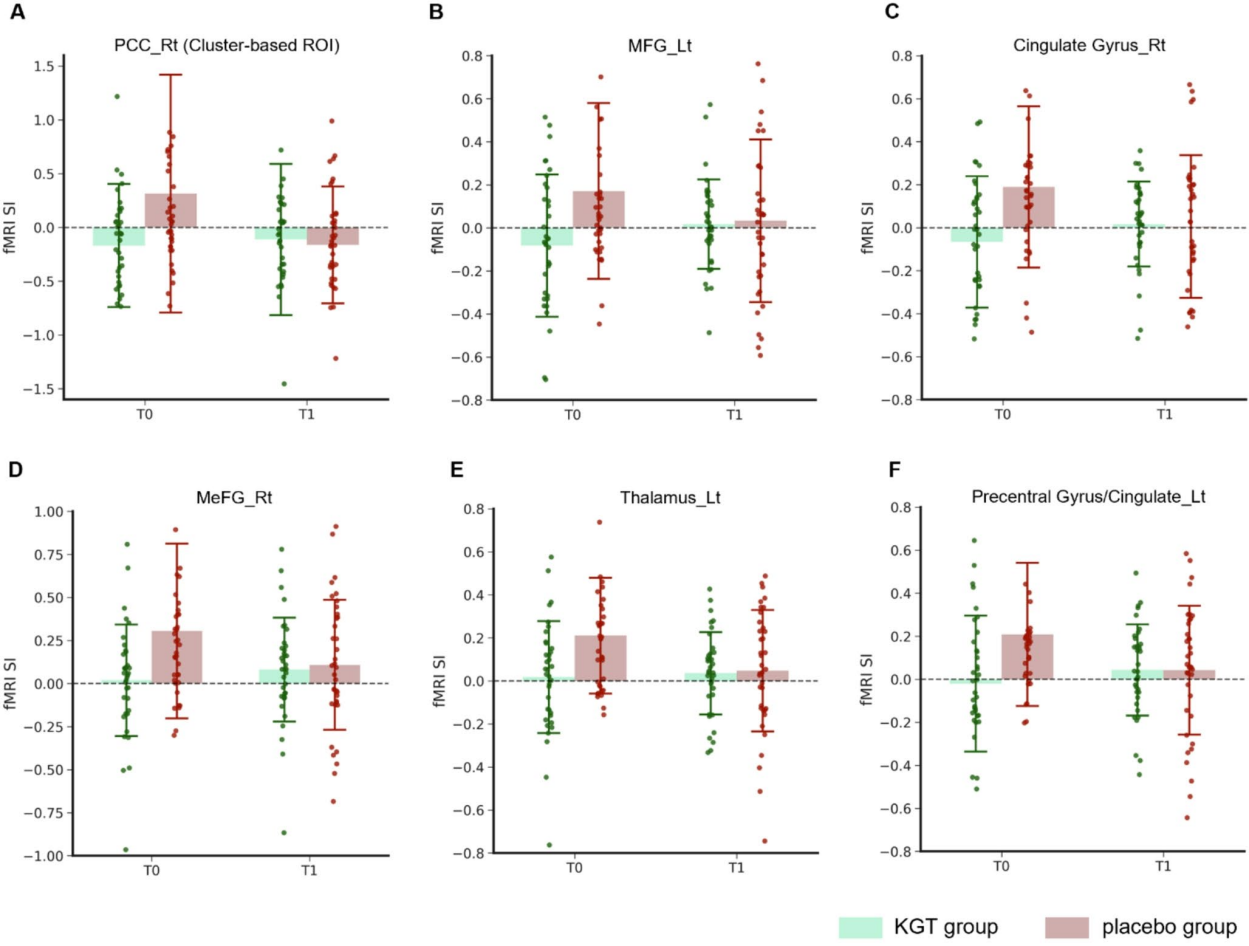
Task-specific ROIs showing significant group × time interaction effects in signal intensity. **A** PCC_Rt identified during the face–name association task. **B**–**F** ROIs demonstrating significant group × time effects during the N-back working memory task: Bars represent group means, and dots indicate individual participants at baseline (T0) and after the 24-week intervention (T1). Error bars show ±1 standard deviation. ROI, region of interest; SI, signal intensity; PCC, posterior cingulate cortex; MFG, middle frontal gyrus; MeFG, medial frontal gyrus; Rt, right; Lt, left.

Across all significant ROIs, the interaction effects were driven by a consistent pattern: the placebo group showed decreases in activation from baseline to 24 weeks, whereas the KGT group showed relative stability or slight increases (ΔKGT ≥ 0 vs. ΔPlacebo < 0). This resulted in reduced between-group differences at follow-up and, in some regions, partial convergence of activation patterns over time (Fig. 4).

### Associations between changes in ROI activation and cognitive performance

Linear regression analyses examining the relationships between changes in ROI activation and changes in SNSB cognitive domain scores identified several significant associations (Supplementary Table S4). In the face–name task, activation in the right precuneus, left PCC, and left precentral gyrus showed negative relationships with language performance in the KGT group. In the placebo group, activation in the right VLPFC was negatively related to visuospatial performance, whereas activation in the left lentiform nucleus was positively related to the same domain. Activation in the right MeFG and left precentral/cingulate region was negatively related to language scores.

In the N-back task, activation in the left lentiform nucleus was positively related to language or executive function in the KGT group, while activation in the left precentral gyrus, left MeFG, and left MFG were negatively related to total SNSB, visuospatial, or memory performance. In the placebo group, activation in the left PHG and right PCC was positively related to memory and attention scores.

### ROI-wise FC analysis

**Fig. 5:**
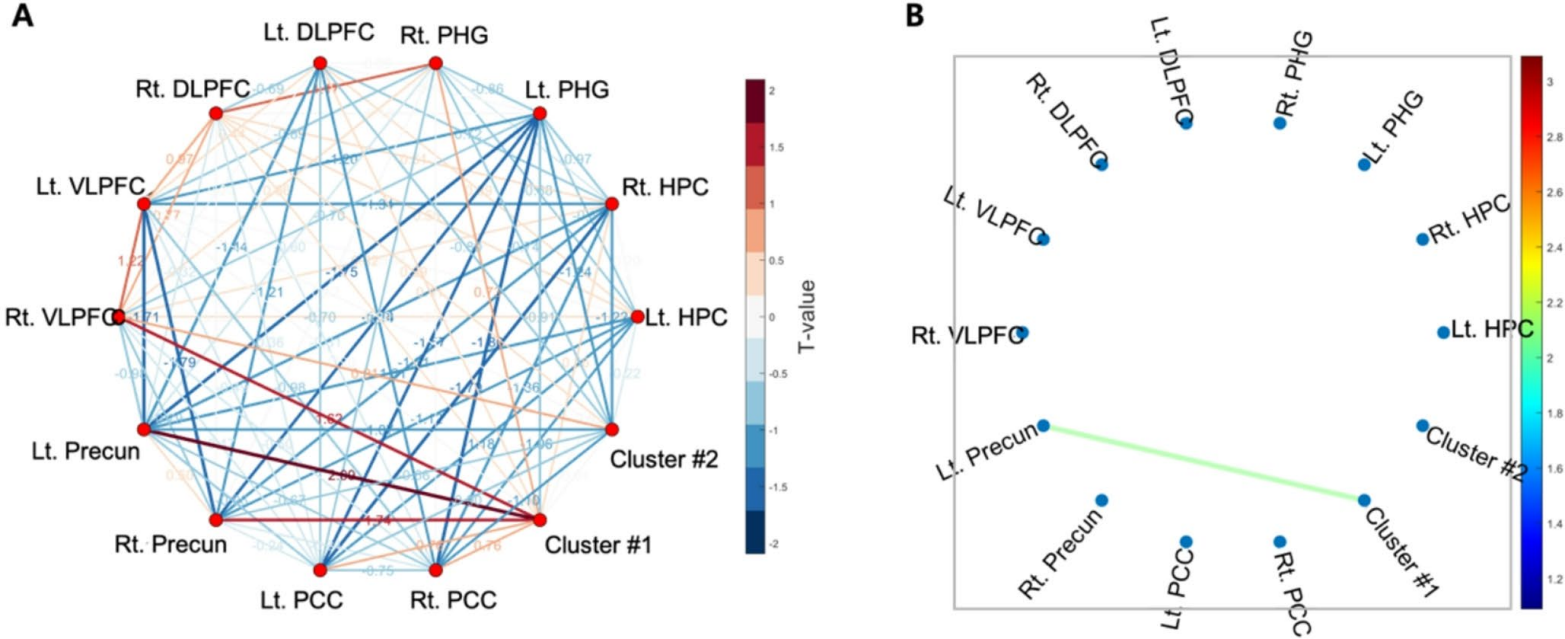
ROI-wise functional connectivity changes within the KGT group. **A** Functional connectivity network across all seeds without a p-value threshold. **B** Significantly increased connectivity between the left precuneus and cluster #1 (right PCC) (*p* = 0.044). Color bars reflect T-values. DLPFC, dorsolateral prefrontal cortex; VLPFC, ventrolateral prefrontal cortex; Precun, precuneus; PCC, posterior cingulate cortex; HPC, hippocampus; PHG, parahippocampal gyrus

FC analysis revealed a significant increase in positive connectivity between the left precuneus and right PCC (cluster-based ROI) in the KGT group (*p* = 0.044; Fig. 5). No significant changes were observed in the placebo group, and no significant between-group differences in FC were found at baseline or post-intervention.

### fMRI task performance and clinical cognitive outcomes

Table 5 summarizes the changes in N-back task performance and MMSE-K scores over the 24-week intervention period. Linear mixed-effects models revealed no significant group × time interaction effects for N-back performance, including accuracy and reaction time in both the 0-back and 1-back conditions. Although small numerical changes were observed within each group, these fluctuations did not reach statistical significance in the LMM analyses.

**Table 5:** fMRI task performance and K-MMSE outcomes showing group × time interaction results

Outcomes	KGT group (*n* = 36) (mean ± SD)		Placebo group (*n* = 37) (mean ± SD)		ΔKGT mean	ΔPlacebo mean	β (Group×Time)	95% CI for β	*p* for Group x Time
	Baseline	24th week	Baseline	24th week					
fMRI task performance									
0-back accuracy (%)^a^	96.34 ± 5.05	95.62 ± 6.54	96.95 ± 4.20	96.07 ± 6.00	–0.98	–1.11	0.101	–3.025, 3.226	0.949
1-back accuracy (%)^a^	93.40 ± 15.00	96.05 ± 6.86	94.57 ± 7.90	96.82 ± 5.59	2.42	2.25	0.3	–5.267, 5.866	0.915
0-back reaction time (ms)^b^	658.81 ± 128.64	621.88 ± 94.39	578.01 ± 118.53	604.55 ± 117.67	–38.81	45.82	–67.576	–138.398, 3.246	0.061
1-back reaction time (ms)^b^	666.95 ± 158.80	624.85 ± 147.93	615.53 ± 226.33	562.81 ± 121.40	–38.68	–39.88	11.519	–90.294, 113.332	0.822
Clinical cognitive outcome									
K-MMSE score	26.89 ± 2.70	27.50 ± 2.47	26.86 ± 2.00	27.61 ± 2.30	0.61	0.67	–0.083	–1.027, 0.860	0.861

Statistical significance was evaluated using linear mixed-effects models assessing group × time interaction effects

*KGT* Kami-guibi-tang, *K-MMSE* Korean version of the Mini-Mental State Examination

^a^ Data available for 34 in KGT group, 35 in Placebo group, ^b^ Data available for 29 in KGT group, 29 in Placebo group

Similarly, K-MMSE scores did not show a significant group × time interaction, with scores remaining generally stable across both groups throughout the study period. Together, these findings indicate that behavioral task performance and global cognitive scores did not exhibit differential longitudinal patterns between the KGT and placebo groups during the 24-week intervention.

## Discussion

In this randomized, double-blind, placebo-controlled trial, we aimed to explore the neural effects of KGT in patients with aMCI, using fMRI. Several findings suggested that KGT may modulate brain function in ways relevant to early cognitive decline; however, these results should be interpreted with caution given the exploratory nature of the study.

Voxel-wise analyses revealed higher baseline activation in the placebo group across both tasks. During the face–name association task, the placebo group showed greater activation in the right PCC. In the N-back task, the placebo group similarly exhibited higher activation in several frontal, cingulate, and subcortical regions, including the lentiform nucleus, inferior frontal gyrus (IFG), middle frontal gyrus (MFG), precentral gyrus, and thalamus. At the 24-week follow-up, however, no significant between-group differences were observed, and the prominent baseline contrasts were no longer present. These descriptive findings should be interpreted with caution, as voxel-wise analyses do not directly assess longitudinal change and are less sensitive than ROI-based approaches for detecting modest temporal effects.

ROI-based linear mixed-effects models revealed significant group × time interaction effects in several regions central to memory and cognitive control. During the face–name task, the right PCC demonstrated a significant interaction, with activation declining in the placebo group but remaining stable in the KGT group. The PCC is a core hub of the default mode network (DMN) and plays a key role in episodic memory retrieval and self-referential processing (Buckner et al., 2008; Cabeza et al., 2008), suggesting that KGT may help maintain engagement of memory-related posterior midline structures during associative encoding attempts.

During the N-back task, interaction effects were observed in the left MFG, right cingulate gyrus, right medial frontal gyrus, left thalamus, and a left precentral/cingulate cluster. These regions participate in working memory updating, attentional allocation, executive control, and cortico-thalamic integration (Barbey et al., 2013; Middleton & Strick, 2000). The left MFG, a component of the dorsolateral prefrontal cortex, contributes to strategic memory processing and flexible manipulation of information (Barbey et al., 2013; Blumenfeld & Ranganath, 2007; Petrides, 2005). The medial frontal and cingulate regions play roles in performance monitoring, conflict detection, and sustained attentional engagement (Etkin et al., 2011; Ridderinkhof et al., 2004). The thalamus serves as a cognitive gate, regulating prefrontal access to task-relevant sensory information and supporting coordinated working memory operations (Halassa & Kastner, 2017; Saalmann et al., 2012). Across all significant ROIs, the placebo group exhibited progressive decreases in activation, whereas the KGT group showed relative stability or slight increases, resulting in partial convergence of activation patterns by follow-up. Importantly, these interaction effects were therefore largely driven by longitudinal decreases in the placebo group rather than by marked increases in activation in the KGT group, raising the possibility that the observed effects reflect differential trajectories over time rather than a definitive upregulation of neural activity induced by KGT.

Collectively, these ROI findings provide a functional context for the voxel-wise observations: although placebo participants demonstrated higher activation at baseline, the longitudinal trajectories—not initial group differences—account for the significant interaction effects. The baseline imbalance may reflect demographic and vascular factors, such as the higher prevalence of hypertension in the KGT group (Iadecola & Gottesman, 2019). Although the between-group difference in age did not reach statistical significance, age is a critical determinant of cognitive function and task-related brain activation, and the slightly higher mean age in the KGT group may also have contributed to the lower baseline activation levels observed in this group. LMMs account for such intercept differences, enabling interpretation centered on differential change rather than baseline contrasts. Given that recall performance was not measured, the face–name activation findings should be interpreted cautiously and viewed as reflecting encoding attempts rather than successful memory formation.

Exploratory analyses relating changes in ROI activation to changes in cognitive performance showed several region-specific associations, though these patterns were inconsistent across tasks and domains. These findings suggest that some neural alterations may accompany cognitive changes, but they do not provide direct evidence of a treatment-specific effect and should be interpreted cautiously.

Resting-state FC analysis revealed a significant increase in connectivity between the left precuneus and the right PCC in the KGT group post-treatment. Both regions are key hubs of the DMN that support episodic memory retrieval and self-related thoughts (Greicius et al., 2004; Wang et al., 2006). DMN disruptions are a hallmark of aMCI and early AD, typically presenting as reduced connectivity between core regions. The observed enhancement in precuneus–PCC connectivity may reflect a partial restoration of DMN coherence, which is consistent with prior studies showing that both pharmacological and non-pharmacological interventions can modulate resting-state networks in MCI (Dunn et al., 2014; Kim et al., 2019).

With regard to behavioral performance, neither N-back accuracy nor reaction time showed notable changes over the 24-week period in either group. Although the KGT group exhibited slower reaction times than the placebo group in the 0-back condition at baseline, this difference did not translate into distinct post-intervention patterns, and no between-group differences were observed in the 1-back condition. Overall, behavioral performance on this low-load working memory task remained stable throughout the study. Global cognitive function, assessed using the MMSE-K, also remained largely unchanged in both groups over the intervention period. The absence of clear behavioral or global cognitive changes is consistent with the exploratory nature of this study and may suggest that the neural differences identified in ROI- and FC-based analyses reflect subtle or early neural modulation that is not readily captured by brief, low-load task measures. Prior studies have reported that neural modulation may precede detectable behavioral change, as shown by training-related hippocampal activation increases in MCI (Hampstead et al., 2012) and by tDCS-induced connectivity changes in older adults (Antonenko et al., 2017).

Taken together, the task-based and resting-state fMRI findings suggest that KGT may influence neural activation and connectivity in regions supporting memory and cognitive control in patients with aMCI. ROI-based analyses showed significant group × time interactions, with activation generally declining in the placebo group but remaining relatively stable in the KGT group across PCC-, frontal-, cingulate-, and thalamic regions. Resting-state analyses further revealed increased precuneus–PCC connectivity in the KGT group. While exploratory, these patterns may reflect subtle or early neural modulation not readily captured by short-term behavioral or cognitive measures. Larger studies with extended follow-up will be needed to determine whether these neural differences translate into clinical benefits.

Several limitations should also be acknowledged. First, baseline group differences—including a higher prevalence of hypertension in the KGT group, an important vascular risk factor, as well as potential age and sex imbalances—may have influenced the results. Second, the face-name task was designed as an encoding-only paradigm, and no recall or recognition data were collected, preventing verification of memory performance and limiting interpretation of task-related activation in relation to successful encoding. Third, the 24-week treatment duration, with only a single post-intervention scan and no long-term follow-up, limits conclusions regarding the persistence of neural effects, particularly given the generally low test–retest reliability of task-based fMRI and resting-state fMRI (Elliott et al., 2020). Fourth, participants were not stratified by aMCI subtype, despite known heterogeneity in clinical progression. Fifth, the 0-back versus 1-back paradigm provides only a modest change in cognitive load, which may have reduced sensitivity to detect treatment-related differences. Sixth, this study relied on region-of-interest and voxel-wise analyses, without applying whole-brain, data-driven methods such as independent component analysis (ICA) or graph-theoretical approaches, which may have limited the scope of network-level interpretations. Future studies should incorporate longer follow-up periods and stratified designs to better assess clinical efficacy. In addition, the inclusion of comprehensive cognitive assessments linked to imaging findings will be essential to clarify whether the observed neural changes are associated with functionally meaningful cognitive benefits. Data-driven approaches could further elucidate the broader network-level mechanisms underlying KGT’s effects.

To the best of our knowledge, this is the first randomized controlled trial to investigate the neural effects of KGT using task-based and resting-state fMRI. Despite its exploratory nature, this study offers novel insight into the potential mechanisms of KGT, supporting its further evaluation in larger, longer-term clinical trials.

## Conclusions

The findings of this study provide preliminary evidence that KGT may modulate brain activity and connectivity in regions associated with episodic and working memory in aMCI. Specifically, ROI-based analyses showed group × time interaction effects in which activation decreased in the placebo group but was preserved or modestly increased in the KGT group across several frontal, cingulate, and thalamic regions, and resting-state analyses demonstrated enhanced precuneus–PCC connectivity following KGT administration. These neural alterations may reflect relative stability of brain activation and connectivity following KGT administration, compared with declines observed in the placebo group.

## Supplementary Information

Supplementary Material 1.

## Data Availability

The raw data supporting the conclusions of this article will be made available by the authors, without undue reservation.
