# Supplementary material for: Effects of the herbal prescription Kami Guibi-tang on brain function in amnestic mild cognitive impairment: a task-based and resting-state fMRI study
Source: Brain Imaging Behav. 2026 Mar 23;20(2):59. doi: 10.1007/s11682-026-01138-6 (PMC13006464; doi:10.1007/s11682-026-01138-6)
Supplement: Supplementary file 1 — Supplementary Material 1. [file 11682_2026_1138_MOESM1_ESM.docx]

Supplementary Material

**Supplementary Information 1.** Full exclusion criteria

1. Diagnosis of dementia according to the NINCDS-ADRDA criteria

2. Diagnosis of vascular dementia according to the NINDS-AIREN criteria

3. Other brain diseases causing neurological symptoms beyond cognitive impairment

4. Degenerative brain diseases such as Parkinson’s disease, Huntington’s disease, Down syndrome, or Creutzfeldt–Jakob disease

5. Brain lesions due to head trauma, hypoxia (e.g., carbon monoxide poisoning), vitamin deficiency, neurosyphilis or encephalitis, brain tumors, endocrine or metabolic diseases, or intellectual disability

6. Clinical evidence of cerebrovascular disease, or suspected territorial infarcts or multiple strokes on MRI

7. History of seizure disorders (excluding childhood febrile seizures)

8. History or current diagnosis of depression, or current use of antidepressants

9. Psychiatric disorders or behavioral problems requiring psychotropic medication, or current use of psychotropic drugs

10. Substance abuse, including drug or alcohol dependence

11. Physical disabilities that are life-threatening and require immediate treatment

12. Uncontrolled hypertension

13. Heart or kidney disease

14. Edema

15. Gastrointestinal symptoms such as anorexia, gastric discomfort, nausea, abdominal pain, or diarrhea

16. Use of medications that may cause hypokalemia or myopathy

17. Hypersensitivity to any component of the investigational drug

18. Women with the possibility of pregnancy

19. Clinically significant abnormalities in blood chemistry (e.g., SGPT/SGOT ≥ 2× upper limit of normal; serum creatinine ≥ 110% of upper limit of normal)

20. Participation in another clinical trial within 4 weeks, or within 5 half-lives of the investigational drug (whichever is longer)

21. Illiteracy

22. Considered unsuitable for participation at the discretion of the investigator

Abbreviations: NINCDS-ADRDA, National Institute of Neurological and Communicative Disorders and Stroke and the Alzheimer’s Disease and Related Disorders Association; NINDS-AIREN, National Institute of Neurological Disorders and Stroke–Association Internationale pour la Recherche et l’Enseignement en Neurosciences; MRI, magnetic resonance imaging; SGPT, serum glutamic pyruvic transaminase; SGOT, serum glutamic oxaloacetic transaminase.

**Supplementary Table S1.** Construction and scoring system for the SNSB-D

| **Domains** | **Score** | **%** | **Subtests** | **Maximum points** |
| --- | --- | --- | --- | --- |
| Attention | 17 | 6 | Digit span forward | 9 |
|  |  |  | Digit span backward | 8 |
| Language and related function | 27 | 9 | Short form of K-BNT (A form) | 15 |
|  |  |  | Calculation  (3 items each for addition, subtraction, multiplication, division) | 12 |
| Visuospatial function | 36 | 12 | RCFT copy | 36 |
| Memory | 150 | 50 | Orientation | 6 |
|  |  |  | SVLT free/delated recalls | 48 |
|  |  |  | SVLT recognition | 12 |
|  |  |  | RCFT immediate/delated recalls | 72 |
|  |  |  | RCFT recognition | 12 |
| Frontal/Executive function | 70 | 23 | Motor impersistence | 3 |
|  |  |  | Contrasting program | 3 |
|  |  |  | Go-no-go test | 3 |
|  |  |  | Fist-edge-palm | 3 |
|  |  |  | Luria loop | 3 |
|  |  |  | Category word generation (animal) | 20 |
|  |  |  | Phonemic word generation (ㄱ) | 15 |
|  |  |  | Stroop test-color reading | 20 |
| GCF score | 300 | 100 |  |  |

K-BNT, Korean-Boston Naming Test; RCFT, Rey-Complex Figure Test; SVLT, Seoul Verbal Learning Test; GCF, Global Cognitive Function.

**Supplementary Table S2.** ROI-based fMRI activation results for the face–name association task from linear mixed-effects models

| **ROI** | **KGT group (n = 36)**  **(mean ± SD)** | |  | **Placebo group (n = 37)**  **(mean ± SD)** | |  | **ΔKGT mean** | **ΔPlacebo mean** | **β (Group×Time)** | **95% CI for β** | ***p* for Group x Time** |
| --- | --- | --- | --- | --- | --- | --- | --- | --- | --- | --- | --- |
|  | **Baseline** | **24^th^ week** |  | **Baseline** | **24^th^ week** |  |  |  |  |  |  |
| Face-name association task | | | | | | | | | | | |
| Hippocampus_Lt. | 0.126 ± 0.21 | 0.212 ± 0.36 |  | 0.259 ± 0.25 | 0.207 ± 0.29 |  | 0.066 | –0.053 | 0.119 | –0.064, 0.302 | 0.200 |
| Hippocampus_Rt. | 0.233 ± 0.46 | 0.243 ± 0.37 |  | 0.247 ± 0.33 | 0.227 ± 0.23 |  | –0.010 | –0.019 | 0.009 | –0.226, 0.244 | 0.937 |
| PHG_Lt. | 0.025 ± 0.29 | 0.142 ± 0.40 |  | 0.160 ± 0.34 | 0.129 ± 0.32 |  | 0.103 | –0.031 | 0.134 | –0.069, 0.336 | 0.192 |
| PHG_Rt. | 0.244 ± 0.53 | 0.242 ± 0.39 |  | 0.215 ± 0.32 | 0.202 ± 0.29 |  | –0.026 | –0.013 | –0.013 | –0.258, 0.233 | 0.918 |
| DLPFC_Lt. | 0.427 ± 0.84 | 0.149 ± 0.58 |  | 0.378 ± 0.62 | 0.277 ± 0.57 |  | –0.286 | –0.100 | –0.186 | –0.588, 0.217 | 0.361 |
| DLPFC_Rt. | 0.451 ± 0.79 | 0.293 ± 0.56 |  | 0.470 ± 0.69 | 0.307 ± 0.57 |  | –0.166 | –0.163 | –0.003 | –0.390, 0.384 | 0.987 |
| VLPFC_Lt. | 0.348 ± 0.77 | 0.251 ± 0.40 |  | 0.383 ± 0.65 | 0.247 ± 0.46 |  | –0.105 | –0.137 | 0.031 | –0.336, 0.399 | 0.865 |
| VLPFC_Rt. | 0.425 ± 0.89 | 0.324 ± 0.53 |  | 0.388 ± 0.67 | 0.237 ± 0.46 |  | –0.116 | –0.151 | 0.035 | –0.373, 0.442 | 0.866 |
| Precuneus_Lt. | 0.148 ± 0.41 | 0.145 ± 0.60 |  | 0.125 ± 0.48 | 0.008 ± 0.57 |  | –0.037 | –0.117 | 0.080 | –0.231, 0.391 | 0.611 |
| Precuneus_Rt. | 0.155 ± 0.42 | 0.136 ± 0.58 |  | 0.104 ± 0.47 | –0.035 ± 0.59 |  | –0.055 | –0.139 | 0.084 | –0.233, 0.402 | 0.597 |
| PCC_Lt. | 0.141 ± 0.42 | 0.136 ± 0.56 |  | 0.109 ± 0.39 | –0.023 ± 0.51 |  | –0.035 | –0.132 | 0.097 | –0.180, 0.375 | 0.487 |
| PCC_Rt. | 0.146 ± 0.42 | –0.031 ± 0.23 |  | 0.112 ± 0.41 | –0.018 ± 0.53 |  | –0.177 | –0.130 | –0.048 | –0.322, 0.226 | 0.729 |
| PCC_Rt. (cluster-based) | –0.168 ± 0.57 | –0.112 ± 0.70 |  | 0.314 ± 1.10 | –0.162 ± 0.54 |  | 0.048 | –0.476 | 0.524 | 0.056, 0.992 | **0.029** |
| Lentiform Nucleus_Lt. | 0.412 ± 0.42 | 0.304 ± 0.46 |  | 0.405 ± 0.5 | 0.387 ± 0.47 |  | –0.136 | –0.018 | –0.118 | –0.413, 0.177 | 0.429 |
| IFG_Lt. | 0.291 ± 0.75 | 0.271 ± 0.37 |  | 0.411 ± 0.84 | 0.245 ± 0.52 |  | –0.032 | –0.166 | 0.134 | –0.269, 0.537 | 0.509 |
| MeFG_Lt. | 0.413 ± 0.81 | 0.254 ± 0.44 |  | 0.374 ± 0.59 | 0.233 ± 0.52 |  | –0.163 | –0.141 | –0.023 | –0.391, 0.345 | 0.902 |
| Precentral Gyrus_Lt. | 0.59 ± 0.5 | 0.563 ± 0.45 |  | 0.745 ± 0.55 | 0.596 ± 0.49 |  | –0.029 | –0.149 | 0.12 | –0.165, 0.405 | 0.403 |
| MFG_Lt. | 0.279 ± 0.77 | 0.125 ± 0.45 |  | 0.311 ± 0.57 | 0.14 ± 0.55 |  | –0.159 | –0.171 | 0.013 | –0.360, 0.385 | 0.946 |
| Cingulate Gyrus_Rt. | 0.163 ± 0.75 | 0.08 ± 0.54 |  | 0.197 ± 0.5 | 0.044 ± 0.47 |  | –0.100 | –0.152 | 0.052 | –0.291, 0.395 | 0.763 |
| MeFG_Rt. | 0.313 ± 0.75 | 0.249 ± 0.51 |  | 0.383 ± 0.69 | 0.218 ± 0.58 |  | –0.089 | –0.165 | 0.076 | –0.281, 0.433 | 0.672 |
| Thalamus_Lt. | 0.292 ± 0.46 | 0.238 ± 0.35 |  | 0.355 ± 0.56 | 0.235 ± 0.52 |  | –0.072 | –0.119 | 0.047 | –0.218, 0.312 | 0.724 |
| Precentral Gyrus/Cingulate_Lt. | 0.166 ± 0.49 | 0.211 ± 0.45 |  | 0.282 ± 0.49 | 0.131 ± 0.42 |  | 0.021 | –0.151 | 0.172 | –0.068, 0.413 | 0.158 |

**Supplementary Table S3.** ROI-based fMRI activation results for the N-back task from linear mixed-effects models

| **ROI** | **KGT group (n = 36)**  **(mean ± SD)** | |  | **Placebo group (n = 37)**  **(mean ± SD)** | |  | **ΔKGT mean** | **ΔPlacebo mean** | **β (Group×Time)** | **95% CI for β** | ***p* for Group x Time** |
| --- | --- | --- | --- | --- | --- | --- | --- | --- | --- | --- | --- |
|  | **Baseline** | **24^th^ week** |  | **Baseline** | **24^th^ week** |  |  |  |  |  |  |
| N-back task | | | | | | | | | | | |
| Hippocampus_Lt. | 0.040 ± 0.25 | 0.083 ± 0.18 |  | 0.119 ± 0.12 | 0.066 ± 0.24 |  | 0.043 | –0.053 | 0.096 | –0.041, 0.233 | 0.168 |
| Hippocampus_Rt. | 0.116 ± 0.23 | 0.243 ± 0.37 |  | 0.070 ± 0.19 | 0.084 ± 0.16 |  | –0.045 | 0.013 | –0.058 | –0.178, 0.063 | 0.344 |
| PHG_Lt. | 0.056 ± 0.24 | 0.100 ± 0.24 |  | 0.088 ± 0.28 | 0.056 ± 0.24 |  | 0.044 | –0.033 | 0.077 | –0.083, 0.238 | 0.341 |
| PHG_Rt. | 0.093 ± 0.21 | 0.085 ± 0.22 |  | 0.103 ± 0.20 | 0.060 ± 0.20 |  | –0.008 | –0.043 | 0.035 | –0.102, 0.171 | 0.612 |
| DLPFC_Lt. | 0.009 ± 0.33 | –0.004 ± 0.23 |  | 0.193 ± 0.40 | 0.073 ± 0.39 |  | –0.013 | –0.119 | 0.107 | –0.118, 0.331 | 0.347 |
| DLPFC_Rt. | 0.080 ± 0.34 | 0.047 ± 0.23 |  | 0.201 ± 0.40 | 0.103 ± 0.43 |  | –0.033 | –0.098 | 0.065 | –0.158, 0.289 | 0.562 |
| VLPFC_Lt. | 0.033 ± 0.31 | 0.075 ± 0.22 |  | 0.223 ± 0.37 | 0.128 ± 0.39 |  | 0.041 | –0.094 | 0.135 | –0.069, 0.340 | 0.190 |
| VLPFC_Rt. | 0.122 ± 0.33 | 0.059 ± 0.27 |  | 0.201 ± 0.35 | 0.132 ± 0.37 |  | –0.063 | –0.069 | 0.007 | –0.211, 0.224 | 0.952 |
| Precuneus_Lt. | –0.002 ± 0.32 | –0.028 ± 0.29 |  | 0.098 ± 0.48 | 0.064 ± 0.38 |  | –0.026 | –0.034 | 0.008 | –0.237, 0.252 | 0.950 |
| Precuneus_Rt. | –0.024 ± 0.33 | –0.033 ± 0.27 |  | 0.090 ± 0.43 | 0.028 ± 0.35 |  | –0.057 | –0.062 | 0.005 | –0.226, 0.236 | 0.965 |
| PCC_Lt. | –0.049 ± 0.33 | –0.038 ± 0.27 |  | 0.049 ± 0.38 | –0.009 ± 0.31 |  | 0.011 | –0.058 | 0.069 | –0.145, 0.282 | 0.524 |
| PCC_Rt. | –0.004 ± 0.28 | –0.031 ± 0.23 |  | 0.049 ± 0.35 | 0.000 ± 0.31 |  | –0.027 | –0.049 | 0.023 | –0.170, 0.215 | 0.815 |
| PCC_Rt. (cluster-based) | –0.043 ± 0.43 | 0.010 ± 0.26 |  | –0.035 ± 0.22 | –0.012 ± 0.36 |  | 0.053 | 0.023 | 0.03 | –0.185, 0.245 | 0.781 |
| Lentiform Nucleus_Lt. | –0.003 ± 0.34 | 0.075 ± 0.24 |  | 0.289 ± 0.44 | 0.138 ± 0.42 |  | 0.078 | –0.151 | 0.229 | –0.008, 0.466 | 0.058 |
| IFG_Lt. | –0.018 ± 0.35 | 0.032 ± 0.2 |  | 0.232 ± 0.34 | 0.124 ± 0.4 |  | 0.05 | –0.108 | 0.159 | –0.057, 0.374 | 0.146 |
| MeFG_Lt. | –0.019 ± 0.27 | 0.038 ± 0.25 |  | 0.211 ± 0.41 | 0.102 ± 0.38 |  | 0.057 | –0.109 | 0.165 | –0.046, 0.376 | 0.122 |
| Precentral Gyrus_Lt. | 0.077 ± 0.27 | 0.128 ± 0.25 |  | 0.304 ± 0.34 | 0.192 ± 0.36 |  | 0.051 | –0.112 | 0.162 | –0.034, 0.359 | 0.104 |
| MFG_Lt. | –0.082 ± 0.33 | 0.018 ± 0.21 |  | 0.171 ± 0.41 | 0.033 ± 0.38 |  | 0.1 | –0.138 | 0.238 | 0.013, 0.463 | **0.039** |
| Cingulate Gyrus_Rt. | –0.066 ± 0.31 | 0.017 ± 0.2 |  | 0.189 ± 0.38 | 0.005 ± 0.33 |  | 0.084 | –0.184 | 0.267 | 0.064, 0.471 | **0.011** |
| MeFG_Rt. | 0.019 ± 0.32 | 0.081 ± 0.3 |  | 0.306 ± 0.51 | 0.109 ± 0.38 |  | 0.062 | –0.197 | 0.259 | 0.039, 0.480 | **0.022** |
| Thalamus_Lt. | 0.018 ± 0.26 | 0.035 ± 0.19 |  | 0.211 ± 0.27 | 0.048 ± 0.28 |  | 0.018 | –0.163 | 0.181 | 0.014, 0.348 | **0.035** |
| Precentral Gyrus/Cingulate_Lt. | –0.02 ± 0.32 | 0.043 ± 0.21 |  | 0.209 ± 0.33 | 0.043 ± 0.3 |  | 0.063 | –0.166 | 0.229 | 0.041, 0.417 | **0.018** |

KGT, Kami-guibi-tang; SI, Signal Intensity; ROI, Region of interest; PHG, Parahippocampal gyrus; DLPFC, Dorsolateral prefrontal cortex; VLPFC, Ventrolateral prefrontal cortex; PCC, Posterior cingulate cortex; IFG, inferior frontal gyrus; MeFG, medial frontal gyrus; MFG, middle frontal gyrus.

Descriptive values are presented as mean ± standard deviation. Statistical significance was evaluated using linear mixed-effects models assessing group × time interaction effects. Values in bold indicate statistical significance (*p* < 0.05).

**Supplementary Table S4.** Significant associations between changes in ROI activation and SNSB cognitive scores (total and domain-specific)

| **Group** | **ROI** | **SNSB Domain** | **95% CI for β** | ***p*-value** |  |
| --- | --- | --- | --- | --- | --- |
|  |  |  |  |  |  |
| Face-name association task | | | | | |
| KGT | PCC_Lt. | Language score | -0.121 (-0.237, -0.006) | 0.041 |  |
|  | Precuneus_Rt. | Language score | -0.126 (-0.248, -0.004) | 0.043 |  |
|  | Precentral Gyrus_Lt. | Language score | -0.145 (-0.255, -0.035) | 0.012 |  |
| Placebo | VLPFC_Rt. | Visuospatial score | -0.066 (-0.127, -0.004) | 0.037 |  |
|  | Lentiform Nucleus_Lt. | Visuospatial score | 0.062 (0.001, 0.124) | 0.046 |  |
|  | MeFG_Rt. | Language score | -0.192 (-0.350, -0.035) | 0.018 |  |
|  | Precentral Gyrus/Cingulate_Lt. | Language score | -0.109 (-0.216, -0.002) | 0.045 |  |
| N-back task | | | | | |
| KGT | Precentral Gyrus_Lt. | Total score | -0.006 (-0.011, -0.001) | 0.029 |  |
|  | Precentral Gyrus_Lt. | Visuospatial score | -0.043 (-0.073, -0.013) | 0.006 |  |
|  | MeFG_Lt. | Visuospatial score | -0.044 (-0.079, -0.010) | 0.013 |  |
|  | Lentiform Nucleus_Lt. | Language score | 0.099 (0.026, 0.171) | 0.009 |  |
|  | Lentiform Nucleus_Lt. | Frontal/Executive score | 0.017 (0.004, 0.030) | 0.011 |  |
|  | MFG_Lt. | Memory score | -0.011 (-0.020, -0.002) | 0.021 |  |
|  | MFG_Lt. | Memory score | -0.010 (-0.020, -0.000) | 0.04 |  |
| Placebo | PHG_Lt. | Memory score | 0.013 (0.000, 0.026) | 0.046 |  |
|  | PCC_Rt. (cluster-based) | Attention score | 0.107 (0.021, 0.193) | 0.016 |  |

KGT, Kami-guibi-tang; ROI, Region of interest; SNSB, Seoul Neuropsychological Screening Battery Dementia version; PCC, posterior cingulate gyrus; VLPFC, Ventrolateral prefrontal cortex; MeFG, medial frontal gyrus; MFG, middle frontal gyrus; PHG, parahippocampalgyrus; Rt, right; Lt, left.

Regression coefficients (β) and 95% confidence intervals were obtained from linear regression models. Only statistically significant associations (*p* < 0.05) are reported.
